# Discrete-Time Visual Servoing Control with Adaptive Image Feature Prediction Based on Manipulator Dynamics

**DOI:** 10.3390/s24144626

**Published:** 2024-07-17

**Authors:** Chenlu Liu, Chao Ye, Hongzhe Shi, Weiyang Lin

**Affiliations:** Research Institute of Intelligent Control and Systems, Harbin Institute of Technology, Harbin 150001, China; chenluliu.cll@gmail.com (C.L.); yechao@hit.edu.cn (C.Y.); hongzheshi.hzs@gmail.com (H.S.)

**Keywords:** discrete-time system, visual servoing control, adaptive prediction, manipulator

## Abstract

In this paper, a practical discrete-time control method with adaptive image feature prediction for the image-based visual servoing (IBVS) scheme is presented. In the discrete-time IBVS inner-loop/outer-loop control architecture, the time delay caused by image capture and computation is noticed. Considering the dynamic characteristics of a 6-DOF manipulator velocity input system, we propose a linear dynamic model to describe the motion of a robot end effector. Furthermore, for better estimation of image features and smoothing of the robot’s velocity input, we propose an adaptive image feature prediction method that employs past image feature data and real robot velocity data to adopt the prediction parameters. The experimental results on a 6-DOF robotic arm demonstrate that the proposed method can ensure system stability and accelerate system convergence.

## 1. Introduction

Vision perception-based robot control is of great significance in intelligent robots. In the fields of autonomous mobile robots [1,2,3,4], humanoid robots [5,6], aerial vehicles [7,8], etc., visual perception is used to improve robot autonomy, environmental adaptability, and intelligence. In addition, in the field of industrial applications, 6-DOF (six degrees of freedom) manipulators with visual servo control are widely used for tasks such as grasping, inserting, and assembling [9,10,11]. There are three visual servo control schemes reported in the literature, namely the (1) position-based visual servoing scheme (PBVS), (2) the image-based visual servoing scheme (IBVS), and (3) the hybrid visual servoing scheme (HVS) [12,13,14]. In the PBVS scheme, the features of the target object are associated with the geometric model of the object to calculate its pose. On the contrary, IBVS does not estimate the pose of the target object but directly uses image features on its controller to control robot’s velocity. Therefore, it does not require precise camera calibration and geometric object models [13,15].

In classical IBVS, the points, lines, and regions of objects are often employed as image features due to their simple recognition and robustness to image noise [16,17]. Then, the controller algorithm is designed using the image Jacobian matrix to convert the image error into the desired robot motion velocity to calculate the control signal. Generally, the proportional control law is employed in classical IBVS to complete the positioning tasks [18]. Although these methods are applicable to practical visual servo tasks, they rely on the geometric features of the object, and the calculation of their Jacobian matrix requires the depth value of the target object. In addition, these methods may cause singularity problems with the image Jacobian matrix [19]. In order to apply the IBVS method to scenes where the target object is prone to deformation, many researchers have explored new image features. Image moments offer a way to describe image features without relying on the geometric characteristics of the target object. Therefore, visual servo methods based on image moments have been widely studied. F. Chaumet proposed a visual servo method based on the general features of image moments [20]. In this study, the interaction matrices of various analytical forms based on image moments were determined. Furthermore, O. Tahri used a normalization method to process image moments and achieved decoupling control of the system by combining the scale-invariant image moments [21]. Another method called direct visual servoing, which uses features such as image histograms [22], Gaussian mixtures [23,24], and wavelets [25], has also received attention from some scholars. To avoid calculating object depth values, Liu et al. [26] constructed a Jacobian matrix that no longer requires depth information by extracting the depth variable from the interaction matrix. Ali et al. [27] proposed a depth-free IBVS method that uses the area of the power port to represent the depth information. To solve the singularity problems, many studies have been devoted to image moment-based visual servoing methods [28,29].

In addition to designing image features, extensive studies have also been conducted on visual servo controllers to achieve better control performance. Xie et al. [9] proposed a switching controller to keep image features within the view range of the camera. Li et al. [30] presented an enhanced controller based on a hybrid proportional derivative control and sliding mode control (PD-SMC) method to ensure that the robot could quickly track target objects and handle uncertainties in depth. Furthermore, considering limitations in terms of the robot’s workspace and velocity, in addition to visibility constraints and parameter uncertainties, visual servoing methods based on model predictive control (MPC) have been proposed [31]. Allibert et al. [32] formulated the constraints as state constraints, as well as input and output constraints. In Hajiloo’s study [33], a robust model predictive control-based IBVS controller was presented, which reduces computational costs and enables MPC to be implemented in a fast system.

Although the above methods solve the problems of classical IBVS by designing new features and different controllers, most of them are designed in continuous-time cases without considering discrete-time system performance [34]. In addition, since robots employed in IBVS are not ideal rigid systems, their dynamic models should also be considered. This will benefit us for predicting image features in discrete-time visual servoing systems. Conticelli et al. derived an interaction model between a camera and an object in discrete time and implemented positioning tasks by designing a visual controller in discrete time [35]. Bjerkeng et al. [36] studied the stability of robot control based on sensor date feedback in discrete time. The authors derived the stability results of visual servoing considering the input/output time delays and the robot dynamics. The result of small-gain proportional control was obtained for the high-delay problem of sensor feedback. He [37] designed an input-mapping method to achieve stability in a discrete visual servo system. Past image errors and camera motion data were employed to calculate the uncertain part of the model. Although these methods have developed visual servo systems from a discrete perspective and designed controllers to ensure system stability, they have not improved system performance by increasing the frequency of image information feedback.

Unlike continuous visual servo methods, this paper describes a visual servo system in discrete time. Furthermore, by analyzing the inner–outer loop control architecture of a visual servo system, the influence of sampling frequency is presented. In order to improve the performance of discrete visual servo systems, this paper develops a discrete-time visual servo controller with adaptive image feature prediction. In the proposed method, we consider both image processing delay and robot dynamics in the system. Past image feature data and robot end-effector velocity data are used to adaptively adjust predictor parameters. The main contributions of this study are summarized as follows:

(1) We present a visual servoing method from the perspective of discrete-time analysis. Based on the visual servo inner–outer loop control architecture, the impact of low-frequency visual feedback on system performance is analyzed.
(2) A linear dynamic model is investigated to describe the movement of the robot under the velocity control mode. By using this model, the actual velocity of the robot end effector can be more accurately estimated.
(3) An adaptive image feature prediction method is proposed to predict the positions of image features during the image sampling and processing time. Past image feature data and real robot velocity data are employed to adjust the predictor parameters.

The rest of this paper is organized as follows. Section 2 describes the discrete-time visual servo model and the problem in a practical discrete-time visual servoing system. Section 3 presents the adaptive image feature prediction based on the liner dynamics model. Section 4 reports the experimental results. Finally, the conclusion is presented in Section 5.

## 2. System Description and Control Architecture

### 2.1. System Description

**Figure 1:**
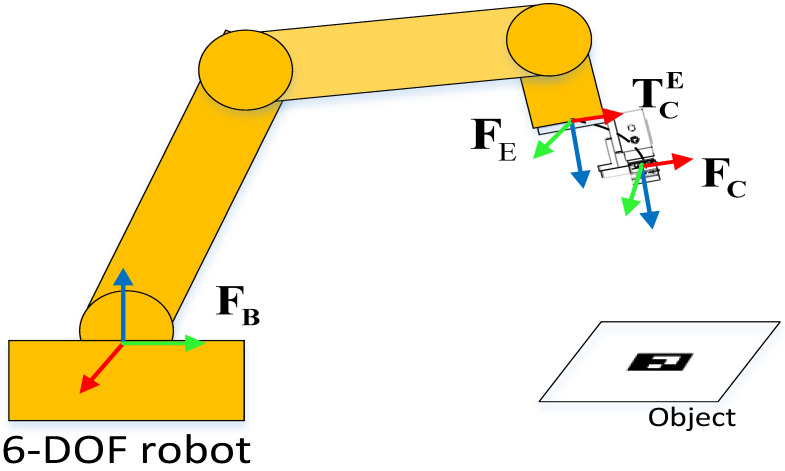
Coordinate system definition in the visual servoing system.

Consider fixing the camera on the end effector of a collaborative robot, as shown in Figure 1. In positioning tasks, the purpose of the IBVS method is to control the robot’s motion until the image features of the object move to the desired feature position in the image, which is typically defined as

(1) $$e\left(t\right)=s\left(t\right)-s^{*},$$

where s(t) is the image feature vector captured by vision sensors at time *t*, and vector $s^{*}$ is the desired value of the features. e∈ϵ is the vector of image error variables, with ϵ being a domain of $R^{m}$. As shown above, in the IBVS scheme, the task error (e) is defined in image space, and the input of the manipulator controller is defined by joint coordinates or Cartesian coordinates. Let $\bar{r}$ be the location of the target object where the features of the object (points, lines, etc.) naturally exist. According to [13], selecting appropriate object features and applying the projection mapping principles, we can obtain

(2) $$s=F(\bar{r}),$$

where F is the perspective projection mapping. Take the derivative of Equation (2) and then the velocities of image features are described as

(3) $$\dot{s}=\frac{\partial s}{\partial \bar{r}}\frac{d\bar{r}}{dt}=LV_{c},$$

where $\dot{s}$ represents the image feature velocities in the image plane and $V_{c}=[v_{x},v_{y},v_{z},w_{x},w_{y},w_{z}{]}^{⊤}$ is the camera velocity screw with respect to the target object. $L\in R^{k\times 6}$ is generally called the image Jacobian or the interaction matrix [4,13,18]. If the system uses image points as features, L can be described as

(4) $$L=\left[\begin{array}{cccccc} -\frac{\lambda }{z} & 0 & \frac{u}{z} & \frac{uv}{\lambda } & -\frac{\lambda^{2}+u^{2}}{\lambda } & v\;\\ 0 & -\frac{\lambda }{z} & \frac{v}{z} & \frac{\lambda^{2}+v^{2}}{\lambda } & -\frac{uv}{\lambda } & -u \end{array}\right],$$

where *z* is the height of image features along the optical axis in the camera coordinates, and λ is the focal length of the camera lens. *u* and *v* are the positions of image features in the image space [13].

In a discrete-time system, the image feature position at time *k* is given by s(k). Let us set the camera sample time as $T_{i}$, and the continuous time is given by $t=kT_{i}$. For simplicity, the following formula uses *k* instead of $kT_{i}$ to represent the current time. To obtain the discrete-time model, Equation (1) is expressed as

(5) $$e\left(k\right)=s\left(k\right)-s^{*}$$

Furthermore, with Equations (3) and (5), the discrete-time model can be obtained as

(6) $$e\left(k+1\right)=e\left(k\right)+TL\left(k\right)V_{c}\left(k\right)$$

To converge image errors to zero, a proportional control scheme is often adopted in classical visual servoing controllers [18], in which

(7) $$\dot{e}\left(k\right)=-K_{p}e\left(k\right)$$

Combining Equations (6) and (7), the camera velocity can be expressed as

(8) $$V_{c}\left(k\right)=-K_{p}L^{+}\left(k\right)e(k),$$

in which $L^{+}$ is the pseudo-inverse of L.

### 2.2. Control Architecture

Defining $q\in R^{n}$ as the robot configuration variables, the IBVS scheme can be expressed as follows:

(9) $$f:q\subseteq R^{n}\mapsto e\subseteq R^{m},e=f\left(q\right)$$

For example, in an IBVS system based on a manipulator, e is the vector of image feature errors, and q is the vector of robot joint position or robot end-effector position.

**Figure 2:**
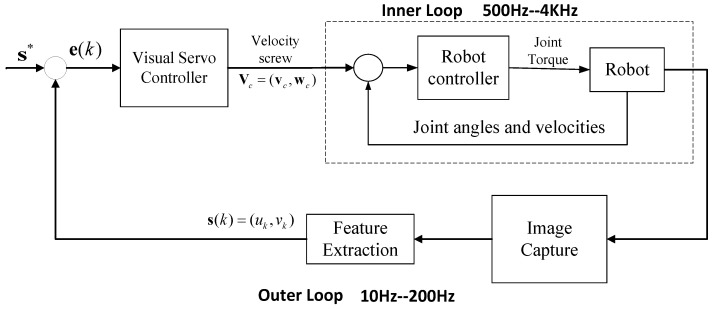
The classical visual servo control architecture.

In IBVS robotic systems, the classic control frame is an inner-loop/outer-loop architecture, as shown in Figure 2. The inner loop is typically composed of a robot controller and robot motor servo drivers. The controller controls the robot’s motion by sending position, velocity, or torque commands to the robot drive. Based on the required speed command, the robot controller uses a robot kinematics or dynamics model to calculate the joint velocities and sends these to the robot. Due to low computational complexity and short transmission delay, the control frequency of the inner loop is usually as high as 500–4000 Hz.

The outer loop, based on image information feedback, executes task layer applications such as grasping, placing, and object tracking. Considering the time required for image data acquisition and processing, the outer loop typically has high computational costs and a low control frequency, typically in the range of 30 Hz to 200 Hz.

**Figure 3:**
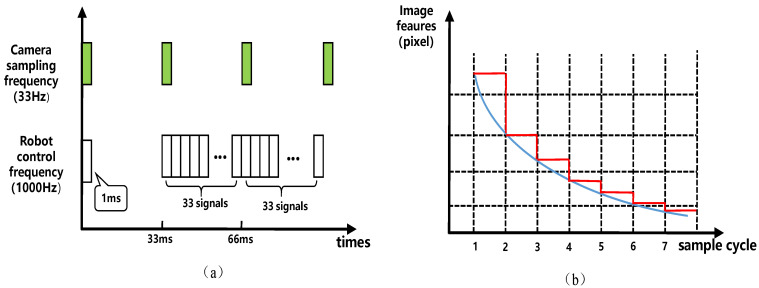
System signals and image feature values. (**a**) Camera sampling and robot control timing diagram. (**b**) Feedback values of image features and actual image features values. Red line: image feature feedback value. Blue line: actual image feature value.

As shown in Figure 3, since the robot control frequency exceeds the image sampling rate, the robot uses the image features of the previous moment during each image feature sampling period until it obtains a new image. $T_{O}$ is the outer-loop cycle time, and $T_{I}$ is the inner-loop cycle time. Therefore, Equation (8) can be rephrased as

(10) $$V_{c}\left(1+(k-1)N\right)=-K_{p}L^{+}e(k),$$

where $N=T_{O}/T_{I}$ and

(11) $$V_{c}\left(1+\left(k-1\right)N\right)=V_{c}\left(m+\left(k-1\right)N\right),$$

in which m=1,2,⋯,N−1, and k=1,2,⋯.

It is noted that the time delay has a great influence on the visual servoing system. The controller uses the same image feature signal within N control cycles, which may lead to system stability and oscillation issues in the worst-case scenarios. Although many scholars have studied image error estimation, they have not explicitly considered the difference between the control period and the image feedback period.

## 3. Adaptive Image Feature Prediction Based on Robot Velocity Linear Dynamic Model

In this section, we introduce the linear dynamic model of the robot end-effector velocity, which benefits us in predicting the image features. Then, the implementation of adaptive image feature prediction is introduced in detail.

### 3.1. Linear Dynamic Model of Robot and Camera Motion

Let frame $F_{E}$ link to the robot end effector and $F_{C}$ link to the camera. The displacement between the robot end-effector coordinate system ($F_{E}$) and the camera coordinate system ($F_{C}$) can be denoted as $P_{C}^{E}=\left(x_{c},y_{c},z_{c}\right)\in R^{3}$, employing $R_{C}^{E}\in R^{3\times 3}$ as the rotation matrix from $F_{C}$ to $F_{E}$. The feature point in the camera coordinate system is denoted as $P_{C}$, and it is represented as $P_{E}$ in the robot end-effector coordinate system, so we have

(12) $$P_{E}=T_{C}^{E}*P_{C},$$

where $T_{C}^{E}$ is the homogeneous transformation matrix, and

(13) $$T_{C}^{E}=\left[\begin{array}{cc} R_{C}^{E} & P_{C}^{E}\\ 0 & 1 \end{array}\right]$$

$V_{e}$ is defined as the robot’s end-effector velocities. Considering the camera is fixedly connected to the robot end effector, the robot end-effector velocities are

(14) $$V_{e}=HV_{c},$$

where

(15) $$H=\left[\begin{array}{cc} R_{C}^{E} & S\left(P_{C}^{E}\right)R_{C}^{E}\\ 0_{3*3} & R_{C}^{E} \end{array}\right],$$

where *S* is the skew symmetric matrix and $S(P_{C}^{E})$ is given by

(16) $$S\left(P_{C}^{E}\right)=\left[\begin{array}{ccc} 0 & -z_{c} & y_{c}\\ z_{c} & 0 & -x_{c}\\ -y_{c} & x_{c} & 0 \end{array}\right]$$

Ideally, the output of the robot’s velocity mode is expected to be a step response, which means that

(17) $${\bar{V}}_{e}\left(n\right)=V_{e}^{ref}\left(n\right),$$

where $V_{e}^{ref}\left(n\right)$ is the expected velocity output by the visual servo controller, and ${\bar{V}}_{e}\left(n\right)$ is the real velocity of the robot end effector.

In practice, the body of a robot is flexible, and its velocity control mode is not ideal. Considering the nonlinear part of its response, we propose a linear model taking into account robot dynamics that is suitable for robots that compute torque in the robot controller [36,38].

Assuming the end velocity of the robot is a uniform acceleration model, within the robot control sample time ($T_{r}$), the robot’s motion distance (*L*) is

(18) $$L=\frac{V_{e}\left(n-1\right)+V_{e}\left(n\right)}{2}t_{1}+V_{e}\left(n\right)t_{2},$$

where $t_{1}$ is the acceleration time, $t_{2}$ is the time for uniform motion, and $T_{r}=t_{1}+t_{2}$. $V_{e}\left(n\right)$ is the actual velocity of the robot. With knowledge of the definition of velocity in physics, the real velocity can be obtained as follows:

(19) $$\begin{array}{cc} {\bar{V}}_{e}\left(n\right) & =L/T\\ \; & =\frac{t_{1}}{2T}V_{e}\left(n-1\right)+\frac{t_{1}+2t_{2}}{2T}V_{e}\left(n\right) \end{array}$$

Let α∈(−1,1) be the convergence rate, employing β as the gain value for the expected velocity. According to Equation (19), the linear dynamic model of the robot velocity is given by

(20) $${\bar{V}}_{e}\left(n\right)=\alpha V_{e}\left(n-1\right)+\beta V_{e}^{ref}\left(n\right)$$

When α=0, the robot velocity controller is perfect and can converge within one step. If the value of α approaches 1, the system is overdamped. For a high-performance robot in velocity control mode, β≈1, indicating that the robot can achieve the required velocity command with a small error. In practice, the robot motion control system is stable. The deviation between the desired velocity and the actual motion velocity is bounded. Thus, we have

(21) $$\left|\right|{\bar{V}}_{e}\left(n\right)||⩽\eta ||V_{e}^{ref}\left(n\right)\left|\right|,$$

where $\eta \in \left(0,\eta_{1}\right),\eta_{1}\in R$.

Currently, most robots provide velocity operation mode in Cartesian space, which greatly facilitates the operation of the robot. Therefore, we can directly send velocity commands to the robot without considering complex joint velocity calculations. According to Equation (14), we have

(22) $${\bar{V}}_{c}\left(n\right)=\alpha H^{+}V_{e}\left(n-1\right)+\beta H^{+}V_{e}^{ref}\left(n\right)$$

### 3.2. Adaptive Image Feature Prediction

In this subsection, we assume that the robot does not have singularity problems. Since the majority of time is spent on image capture and processing in the outer loop, we set $T_{O}=T_{i}$ and $T_{I}=T_{r}$. In most IBVS systems, the time ($T_{i}$) is much longer than the robot controller time ($T_{r}$). Therefore, we use the robot velocity information at $nT_{r}$ to estimate the image error. Considering the model dynamics, according to Equations (14) and (20), we have

(23) $$e\left(n+1\right)=e\left(n\right)+TL\left(n\right)H^{+}{\bar{V}}_{e}\left(n\right)$$

When $n=k(T_{i}/T_{r})$, n,k=1,2,⋯, we can obtain the image error directly from the camera. If $n\neq k(T_{i}/T_{b})$, we employ Equation (23) to estimate the image error.

It is important to accurately update α and β to estimate image feature positions. It should also be noted that due to the uncertainty of robot dynamics, α and β vary with robot posture.

Here, an effective method based on the least squares method is proposed for adaptive adjustment of α and β. The image feature errors and robot end-effector velocities are employed as observational data. In the first control cycle of the robot control inner loop, according to Equation (23), we can obtain

(24) $$e\left(1\right)-e\left(0\right)=T_{r}L\left(1\right)H^{+}\left(\alpha V_{e}\left(0\right)+\beta V_{e}^{ref}\left(1\right)\right)$$

Similarly, the image feature errors are calculated sequentially in other control cycles as

(25) $$\begin{array}{cc} e(2)-e(1) & =T_{r}L\left(2\right)H^{+}\left(\alpha V_{e}\left(1\right)+\beta V_{e}^{ref}\left(2\right)\right)\\ e(3)-e(2) & =T_{r}L\left(3\right)H^{+}\left(\alpha V_{e}\left(2\right)+\beta^{+}V_{e}^{ref}\left(3\right)\right)\\ \; & \;\vdots \\ e(n-m)-e(n-m-1) & =T_{r}L\left(n-m\right)H^{+}\left(\alpha V_{e}\left(n-m-1\right)+\beta V_{e}^{ref}\left(n-m\right)\right) \end{array}$$

Let $F\left(n\right)=T_{r}L\left(n\right)H^{+}$; for the above iterative process, the prediction equation is

(26) $$e\left(n\right)-e\left(n-1\right)=F\left(n\right)(\alpha V_{e}\left(n-1\right)+\beta V_{e}^{ref}\left(n\right)).$$

In order to better represent the parameters of the linear dynamic model of robot end velocity, we set $Z\left(n\right)=F^{-1}\left(n\right)\left[e\left(n\right)-e\left(n-1\right)\right]$ as the system output, and Equation (26) can be rewritten as

(27) $$Z\left(n\right)=\left[\begin{array}{c} V_{e}\left(n-1\right),V_{e}^{ref}\left(n\right) \end{array}\right]\left[\begin{array}{c} \alpha \\ \beta \end{array}\right]$$

Assuming that the length of the moving window is *K* and $\hat{\theta }={\left[\alpha ,\beta \right]}^{T}$, we collect Equations (25) and (26) in a compact matrix form as

(28) $$Z\left(n\right)={\vec{F}}^{-1}Y=\Phi \hat{\theta },$$

where

(29) $$Z\left(n\right)={\left[Z\left(n\right),Z\left(n-1\right),\ldots ,Z\left(n-K+1\right)\right]}^{T}$$

(30) $$\vec{F}={\left[\begin{array}{cccc} F(n) & 0 & \cdots & 0\\ 0 & F(n-1) & \cdots & 0\\ \vdots & \vdots & \vdots & \vdots \\ 0 & 0 & 0 & F(n-K+1) \end{array}\right]}_{K\times K}$$

(31) $$Y={\left[\begin{array}{c} e(n+1)-e(n)\\ e(n)-e(n-1)\\ \vdots \\ e(n-K+2)-e(n-K+1) \end{array}\right]}_{K\times 1}$$

(32) $$\Phi ={\left[\begin{array}{cc} V_{e}\left(n\right) & V_{e}^{ref}\left(n+1\right)\\ V_{e}\left(n-1\right) & V_{e}^{ref}\left(n\right)\\ \vdots & \vdots \\ V_{e}\left(n-K+1\right) & V_{e}^{ref}\left(n-K+2\right) \end{array}\right]}_{K\times 2}$$

We employ $\hat{Z}\left(n\right)$ as the measurement value of the system. When $n=k(T_{i}/T_{r})$, $\hat{Z}\left(n\right)$ is

(33) $$\hat{Z}\left(n\right)=F^{-1}\left(n\right)\left[\hat{e}\left(n\right)-e\left(n-1\right)\right]$$

where $\hat{e}\left(n\right)$ is the actual image error at the image sample time. During the non-sampling period of the camera, let $\hat{Z}\left(n\right)=Z\left(n\right)$. Thus, the measurement value ($\hat{Z}\left(n\right)$) can be expressed as

(34) $$\hat{Z}\left(n\right)=\left\{\begin{array}{cc} F^{-1}\left(n\right)\left[\hat{e}\left(n\right)-e\left(n-1\right)\right] & n=k(T_{i}/T_{r})\\ Z(n) & n\neq k(T_{i}/T_{r}) \end{array}\right.$$

We set $\hat{Z}$ as the vector representation of $\hat{Z}\left(n\right)$ in *K* control cycles. We define the cost function as

(35) $$J=\sum_{n=1}^{K}{\left[\hat{Z}\left(n\right)-Z\left(n\right)\right]}^{2}$$

By using the least squares method, the $\hat{\theta }$ parameter can be obtained as

(36) $$\hat{\theta }={\left(\Phi^{T}\Phi \right)}^{-1}\Phi^{T}\hat{Z}$$

**Figure 4:**
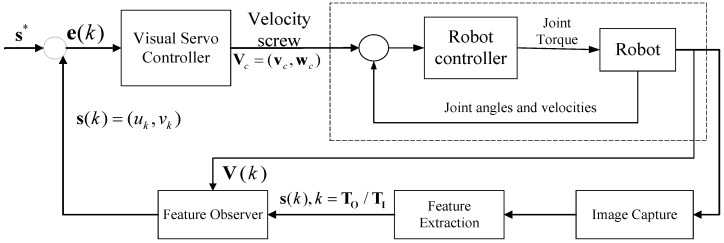
Discrete-time visual servo control system with adaptive image feature prediction.

Figure 4 shows our overall visual servo system. In contrast to basic visual servoing methods, the proposed feature prediction method combines past image data and robot end-effector velocity to predict the current image features. The method steps are given in Algorithm 1.
**Algorithm 1** Visual Servoing Controller With Adaptive Image Feature Prediction**Input:** the robot end-effcetor velocity $V_{e}\left(n\right)$ and the image features e(k) **Output:** the robot end-effector velocity command $V_{e}^{ref}\left(n\right)$;
 1:Set initial state, such as α=0,β=1, the window length *K* ($K>(T_{i}/T_{r})$), the control gain $K_{p}$, the final image feature errors δ, the robot control time n=1 and the image feature sampling time k=1. H is the constant matrix and e(1) can be directly obtained from the camera. 2:Calculate the robot end-effector command $V_{e}^{ref}$ by (8) and (14). 3:If $kT_{i}=nT_{r}$, the image error e is obtained through the camera and k←k+1. Otherwise, the image error e is predicted through (20) and (23). 4:Update the matrix Y and Φ by the past data. If n>K, then calculate the $\hat{\theta }$ by (36). 5:If e>δ, update n←n+1 and come back to Setp. 2.


### 3.3. Stability Analysis

The Lyapunov function is designed as

(37) $$V\left(n\right)=\frac{1}{2}{\left||e\left(n\right)|\right|}^{2}=\frac{1}{2}e^{T}\left(n\right)e\left(n\right)$$

Furthermore, we define ΔV(n)=V(n+1)−V(n). According to Equations (8), (21), and (23), we have

(38) $$\Delta V\left(n\right)=\frac{1}{2}\left(|\right|\left(I-\eta TK_{p}\right)e\left(n\right){\left|\right|}^{2}-||e\left(n\right){\left|\right|}^{2})$$

To ensure system stability, the following condition should be satisfied [37]:

(39) $$||I-\eta T_{r}K_{p}{\left|\right|}^{2}<I$$

In practice, η is close to 1, and T is very small. We can choose the appropriate $K_{p}$ to ensure the system’s stability and improve its performance.

## 4. Experiments

**Figure 5:**
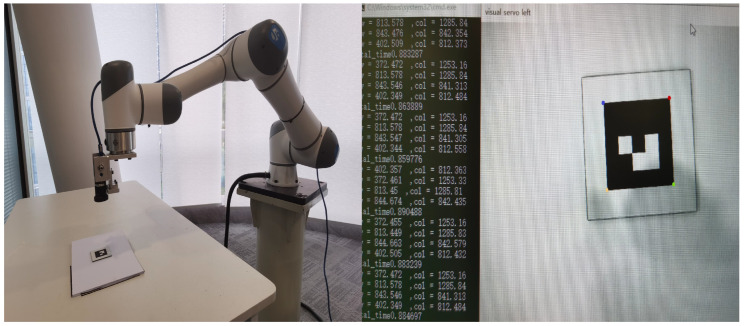
Experimental setup.

Several comparative experiments were designed to demonstrate the impact of the proposed method on system performance. We used a lightweight collaborative robot and a camera fixed on a 6-DOF collaborative robot end effector. When the robot is in velocity mode, the controller can send speed commands to control the movement of the robot’s end effector. We also selected the corner points of the ArUco marker as the feature points. The experimental equipment is shown in Figure 5.

The robot has a velocity control mode with a communication frequency of 1000 Hz. A Galaxy camera is employed to capture the position of the feature points, with a resolution of 1920 × 1080. Considering the image acquisition time and image processing time, the feedback frequency of image features is about 30 Hz. According to the design and calibration parameters, $T_{C}^{E}$ is

(40) $$T_{C}^{E}=\left[\begin{array}{cccc} 0 & 1 & 0 & 0.05\\ -1 & 0 & 0 & 0\\ 0 & 0 & 1 & 0.08\\ 0 & 0 & 0 & 1 \end{array}\right]$$

We conducted comparative experiments of visual servoing using the following three methods:

(1) Method 1 utilized the classical visual servoing scheme (VSC) [18]. In VSC, the four points are employed as image features. Due to the easy recognition of image features, the simple interaction matrix of point features, and high computational efficiency, this scheme has been widely applied in practice.
(2) Method 2 (VSC-OB) employed the image prediction method based on the observer, which only uses Equation (6) and does not consider the robot dynamic model.
(3) Method 3 (VSC-P) utilized the proposed adaptive image prediction based on the input-mapping method in combination with the robot’s dynamic model.

The robot moves from the initial position where the joint angles are (q=[−100.5,29.6,97.1,−36.68,−90.37,−10.26]) and the image points are ((1214,581),(1214,960),(836,960)) and (836,581). The length of the moving window is set as $K=T_{i}/T_{r}=33$. We only considered the translational motion of the robot in the experiments, disregarding the rotational movements.

### 4.1. Controller with Smaller Proportional Gain

In this subsection, a comparative experiment with the controller selecting a small proportional gain is designed to compare the three different methods. In this comparative experiment, the gain is set as 15.

**Figure 6:**
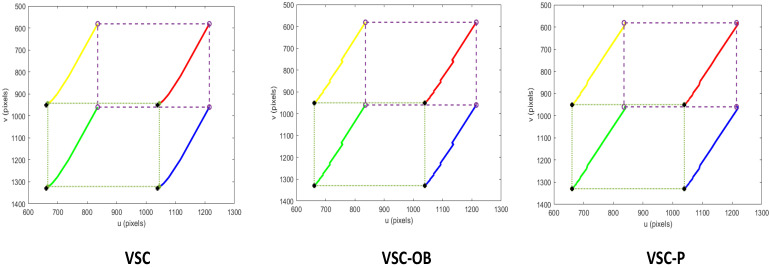
Image trajectory from the start position (∘) to the desired position (+) in image space using a controller with a small proportional gain.

Figure 6 shows the feature trajectory within the image space of the three methods. It can be observed that all the methods are capable of accomplishing the visual servoing task of moving the robot from the start position to the desired location. At a small proportion of gain, the trajectories in the image space are smooth and close to straight lines.

**Figure 7:**
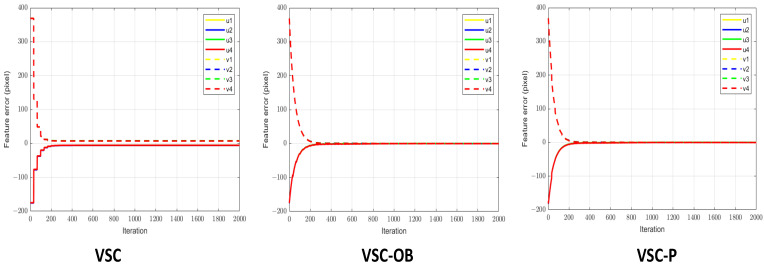
Image feature errors with a small proportional gain controller.

**Figure 8:**
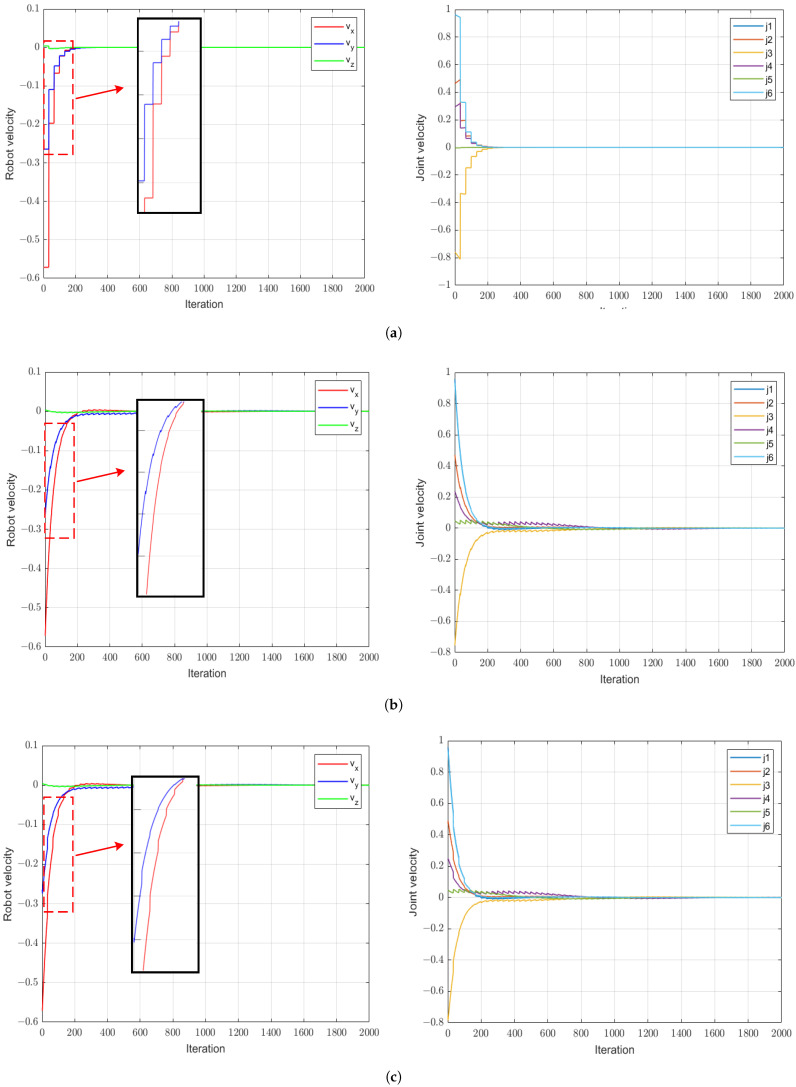
Robot end-effector and joint velocities with a small proportional gain controller. (**a**) VSC method. (**b**) VSC-OB method. (**c**) VSC-P method.

Figure 7 displays the image feature error, and Figure 8 shows the end-effector velocities and joint velocities of the robot. We can see that with the application of a small proportional gain controller, all three visual servo methods can cause the robot’s end-effector velocity to converge. However, due to the low frequency of image information feedback, the VSC scheme exhibits a stepped error in its image features and experiences significant speed fluctuations during the servo process. On the contrary, owing to the presence of the image feature observer, VSC-OB can predict image features and minimize the velocity changes of the robot end effector. VSC-P integrates an image feature observer and considers the robot’s dynamic characteristics, resulting in smoother velocity control at the robot end effector.

### 4.2. Controller with Large Proportional Gain

To explore the control performance of the proposed method with larger proportional gains, a controller with a high proportional gain value is used in this subsection. In this experiment, the proportional gain value is set as 50.

**Figure 9:**
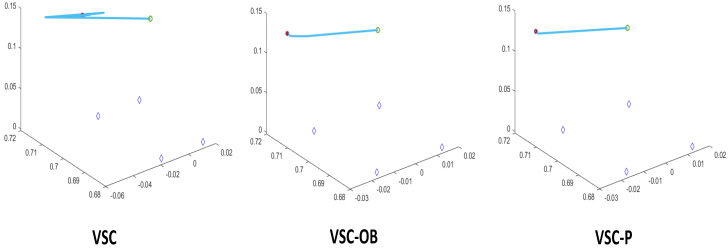
Robot end-effector moving trajectory from the start position (∘) to the desired position (⋇).

**Figure 10:**
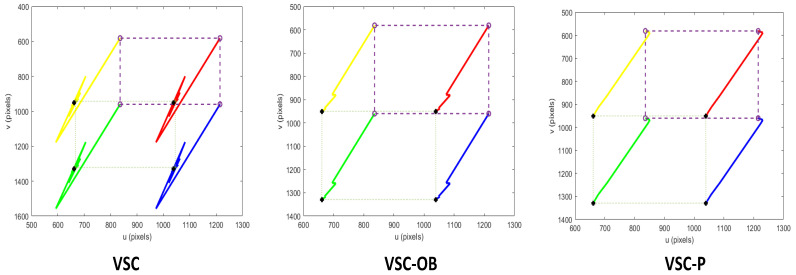
Image trajectory from the start position (∘) to desired position (+) in image space with a large proportional gain controller.

Figure 9 illustrates the moving trajectory of the robot end effector using three different schemes, while Figure 10 shows the feature trajectory in the image space. It can be observed that with a larger proportional gain controller, the VSC method induces vibration as the robot end effector approaches the target position. In the VSC-OB method, the trajectory of the robot end effector experiences a slight jitter. Compared to the first two methods, the proposed method achieves a smoother trajectory for the robot’s motion and ensures that there are no oscillations when the robot approaches the target position.

**Figure 11:**
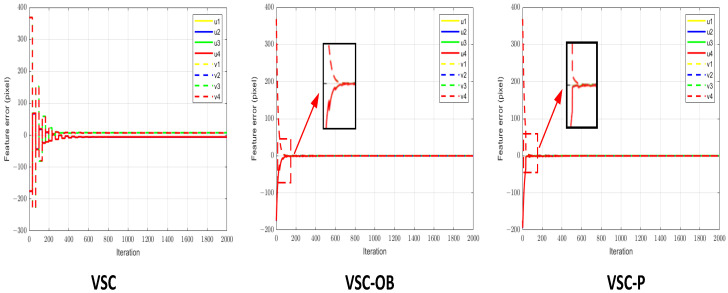
Image feature errors with a large proportional gain controller.

**Figure 12:**
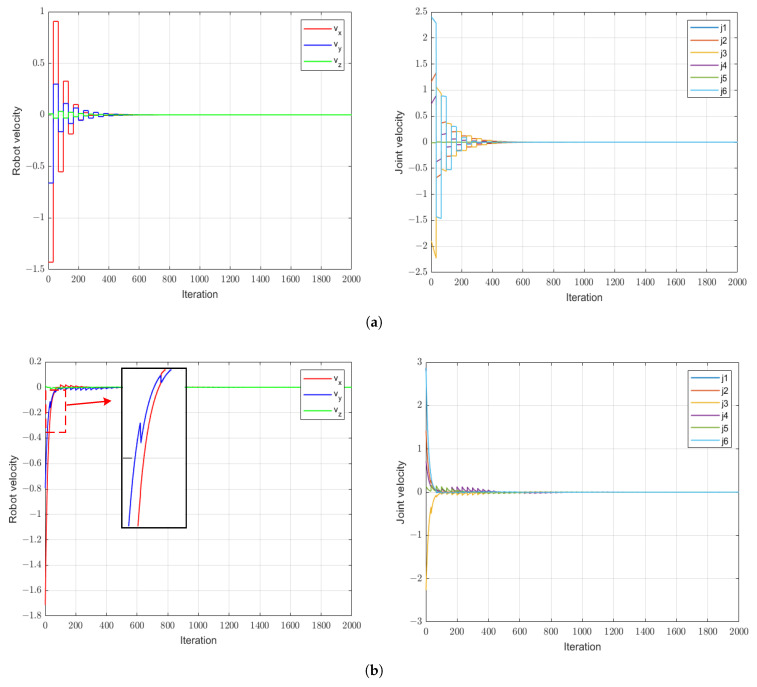

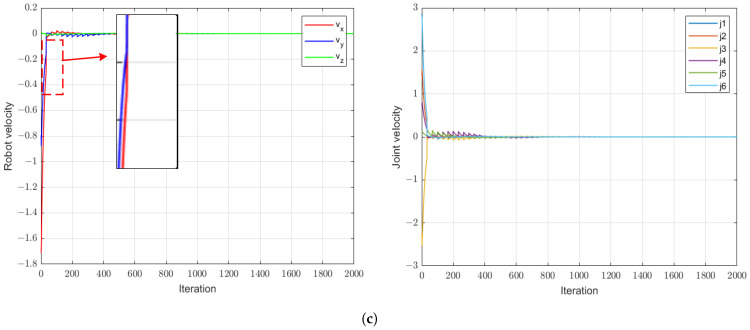
Robot end-effector and joint velocities with a large proportional gain controller. (**a**) VSC method. (**b**) VSC-OB method. (**c**) VSC-P method.

The image errors of the three methods are shown in Figure 11, and the robot end-effector and joint velocities are shown in Figure 12a–c. It is obvious that in the case of large gain values, the VSC scheme causes oscillation of the robot’s end-effector movement during the visual servo process. In contrast, because of the precise prediction of changes in image error during the control cycle, the control signal can decrease as the image error diminishes. Therefore, both the VSC-OB method and the proposed VSC-P method can not only achieve convergence but also improve the convergence speed of the system when employing a larger proportion of controllers. Furthermore, we can see that compared with the VSC-OB method, the proposed method takes into account the dynamic characteristics of the robot’s velocity mode, making image error prediction more accurate. Therefore, the robot’s end-effector motion is smoother than under the VSC-OB method.

## 5. Conclusions

In this paper, a visual servoing control method is investigated from the perspective of a discrete-time model. To solve the time delay issue of the outer loop, image features are directly estimated in the image plane, which is suitable for scenes with low camera sampling frequency and complex image processing. This work designed an adaptive image feature prediction method by using past image feature data and robot end-effector velocity data. The controller with adaptive feature prediction smooths the velocity input of the IBVS system and still maintains the stability of the system under large gains. Compared with the classical IBVS scheme, the VSC-P method is more applicable for situations with a large outer-loop time delay. For scenarios that require high resolution and high-precision positioning, such as industrial assembly, our method can greatly reduce the problem of slow system convergence caused by long image computation time. It should be noted that the proposed method does not take into account cases of image feature feedback errors or image information loss. Therefore, in future work, we will further investigate the estimation of image features at the sampling time to reduce the impact of image feature information loss or image noise on system performance.

## Data Availability

Data are contained within the article.
